# Scientific Advisory Committees at the World Health Organization: A Qualitative Study of How Their Design Affects Quality, Relevance, and Legitimacy

**DOI:** 10.1002/gch2.201700074

**Published:** 2018-08-12

**Authors:** Unni Gopinathan, Steven J. Hoffman, Trygve Ottersen

**Affiliations:** ^1^ Division for Health Services Norwegian Institute of Public Health 0473 Oslo Norway; ^2^ Oslo Group on Global Health Policy Department of Community Medicine and Global Health and Centre for Global Health Institute of Health and Society Faculty of Medicine University of Oslo 0450 Oslo Norway; ^3^ Global Strategy Lab Dahdaleh Institute for Global Health Research Faculty of Health and Osgoode Hall Law School York University Toronto Ontario M3J 1P3 Canada; ^4^ Department of Health Research Methods, Evidence, and Impact and McMaster Health Forum McMaster University Hamilton Ontario L8S 4L8 Canada; ^5^ Department of Global Health and Population Harvard T.H. Chan School of Public Health Harvard University Boston MA 02115 USA

**Keywords:** boundary organizations, CRELE, evidence‐informed health policy, science–policy interface, scientific advice, scientific advisory committees, World Health Organization

## Abstract

Governments and international organizations frequently convene scientific advisory committees (SACs) to support decision‐making with scientific advice. In this study, thematic analysis of interviews with 35 senior WHO staff identified five main themes characterizing WHO's experience with designing SACs to ensure quality, relevance, and legitimacy of scientific advice. First, in addition to technical matters, SACs are established to serve broader strategic objectives, including consensus building to promote high‐level political messages. Second, for SACs to be fully independent, they must have autonomy from the institutions convening or funding them, from the institutions from where SAC members are recruited, and from the institutions to whom the advice is directed. Third, since choices affecting quality, relevance, and legitimacy are closely linked, designing SACs often require trade‐offs among these three attributes. Fourth, staff supporting SACs need to balance between safeguarding SACs from external influence and being receptive to the external political environment. Fifth, the design of SACs need to balance the involvement of stakeholders with the power to act on recommendations against the need to protect the independence and integrity of the scientific process. Overall, this study highlights key choices conveners of SACs must make when seeking to promote quality, relevance, and legitimacy of scientific advice.

## 1 Introduction

Policymakers at all levels of decision‐making commission scientific advice to inform their decisions. To facilitate this process, governments and international organizations frequently convene scientific advisory committees (SACs), which can be understood broadly as a group of individuals with some kind of expertise that provide advice to policymakers informed by evidence from the natural and social sciences. Some SACs are established as time‐limited entities, while others, such as the Intergovernmental Panel on Climate Change, are permanent institutions.^1^

A rich literature has explored the design, role, and influence of SACs in global environmental governance^2^, ^3^, ^4^, ^5^, ^6^ and has clarified different ways of thinking about how the design of SACs can influence their impact. A useful framework is the one initially developed by Cash et al.,^7^, ^8^ and later applied by many others,^2^, ^3^, ^9^, ^10^, ^11^ which proposes that three key attributes influence the effectiveness of SACs: credibility (sometimes used interchangeably with “quality”), salience (sometimes used interchangeably with “relevance”), and legitimacy. A fundamental assumption underlying this framework is that strengthening the quality, relevance, and legitimacy of SACs will enhance their effectiveness.^7^, ^8^ In the context of SACs, a common way of understanding effectiveness is as “the ability to influence the behavior of intended audiences by enhancing their knowledge of the consequences of their decisions.”^2^, ^11^

Similar to global environmental governance, a great number of SACs exist to inform intergovernmental processes and national policymaking on global health issues. To our knowledge, few studies have examined the institutional design and effectiveness of these bodies in this context, despite their common use by governments, international organizations, civil society, businesses, and other entities. The World Health Organization's (WHO) constitution mandates this United Nations (UN) specialized agency “to provide objective and reliable information and advice in the field of human health”—a function that has largely been fulfilled by convening SACs.^12^ Since its establishment in 1948, the agency has produced technical guidelines, norms, standards, and policy recommendations through a range of different SACs, such as expert committees (some of which are as old as the organization itself), guideline development panels, and high‐level commissions (**Table** **1**). Overall, WHO has extensive experience convening SACs to inform policy and practice at the global and national levels. Accordingly, this organization represents a valuable case for drawing wider lessons about how to (and how not to) design SACs to increase quality, relevance, and legitimacy of scientific advice. The aims of this study were to characterize the different types of SACs that exist in WHO's ecosystem and to identify the institutional design features that influence the quality, relevance, and legitimacy of the advice they offer.

**Table 1:** Overview of WHO's various types of scientific advisory committees

**General description** A general classification of SACs in WHO distinguish between expert and nonexpert advisory groups.^13^ Advisory panels, expert committees, study groups, and scientific groups are, according to WHO's nomenclature, considered “expert groups” and must conform to regulations stipulated in WHO's Basic Documents that govern the agency.^14^ Other SACs, such as guideline development groups, strategic advisory groups, and commissions are considered “non‐expert advisory groups” without specific rules of procedure, although the Basic documents stipulate that these mechanisms should be “in general conformity with the principles outlined in these regulations, especially concerning the adequate international and technical distribution of expertise.”^14^ **Overview of SACs in WHO** ***Advisory Panels and Expert Committees*** Advisory panels and expert committees are the only SACs that are formally defined in WHO's Basic Documents.^14^ According to those documents, WHO's Director‐General may establish an expert advisory panel in any field, to be utilized in whatever manner is needed. Expert advisory panels consist of experts from whom WHO regularly obtains technical guidance and support within a particular area, either by correspondence or at meetings to which these experts may be invited. An expert committee is a group of expert advisory panel members convened by the Director‐General for reviewing and making technical recommendations on a subject deemed important for WHO and its member states. The Expert Committees are standing committees where after a period old members can be re‐elected and new members can be recruited. One example is the WHO Expert Committee on Selection and Use of Essential Medicines.^15^ ***Guideline Development Panels*** WHO's guideline development process is outlined in the agency's Handbook on Guideline Development.^16^ A WHO guideline contains recommendations for clinical practice or public health policy that help users of the guidelines “make informed decisions on whether to undertake specific interventions, clinical tests or public health measures, and on where and when to do so, and to help the user to select and prioritize across a range of potential interventions.”^16^ WHO describes a recommendation to tell “the intended end‐user of the guideline what he or she can or should do in specific situations to achieve the best health outcomes possible, individually or collectively,” and to offer “a choice among different interventions or measures having an anticipated positive impact on health and implications for the use of resources.”^16^ Technical departments in WHO initiate and coordinate the guideline development process. Four different groups are established: a steering group; a guideline development panel; an external review group; and a systematic review team. The target audience of guidelines are typically either policymakers, managers in the health sector, or health professionals. WHO's handbook emphasizes that “guidelines must have a clearly defined target audience (end‐user) which is identified early in the guideline development process,” that “the recommendations need to be tailored to that audience,” and that writing guidelines to simultaneously meet the needs of very different audiences—policymakers, health sector managers, clinicians and other health professionals—is difficult and “should be avoided.”^11^ Examples of published guidelines include “WHO recommendations on antenatal care for a positive pregnancy experience,”^17^ “WHO guideline on country pharmaceutical pricing policies,”^18^ and “WHO guidelines on optimizing health worker roles for maternal, newborn, and child health.”^19^ ***Scientific and Technical Advisory Groups*** The formal WHO documents do not define the design and purpose of these advisory bodies. There exist a great deal of variation in the type of Scientific and Technical Advisory Groups (STAGs) convened, with respect to the kind of topics addressed, type of advice that is produced, and the time‐frame over which these bodies operate. WHO has STAGs advising on issues such as tuberculosis,^20^ neglected tropical diseases,^21^ immunization,^22^ nutrition guidance,^23^ among others. Most STAGs are standing committees that advise WHO's departments on priorities and strategic focus, while some focus on developing guidance and policy recommendations addressing specific technical issues. Different STAGs vary with respect to how detailed these have described their process for developing evidence‐informed guidance. The Strategic Advisory Group of Experts (SAGE) on Immunization have, for example, developed their own document for “Guidance for the development of evidence‐based vaccination‐related recommendations.”^24^ ***Technical Meeting Reports*** WHO frequently convenes scientists and other stakeholders on an ad‐hoc basis for meetings to review the latest evidence on specific health issues, which often result in the publication of meeting reports containing policy recommendations. These meetings are not intended to result in formal WHO recommendations. However, occasionally, the advice from such meetings is promoted as if they were official WHO technical advice and recommendations. A notable example is the 2016 Technical Meeting Report on Fiscal Policies for Diet and Prevention of Noncommunicable Diseases, which included a widely promoted recommendation from WHO to implement a tax on sugar‐sweetened beverages.^25^ ***High‐Level Commissions*** WHO's Director‐General may convene a group of experts to comprehensively review and analyze the status of a topic and propose recommendations that can support WHO and its member states in addressing the issue. Commissions typically spend a longer time than other SACs before concluding their work. Previous commissions include the Commission on Social Determinants of Health^26^ and the Commission on Macroeconomics and Health.^27^ ***Special committees, expert working groups, and other types of SACs established after endorsement by WHO member states*** WHO's Executive Board or its World Health Assembly, at the initiative of WHO's Director‐General or member states themselves, adopt resolutions that request the establishment of committees, expert working groups, or other forms of SACs to review and assess specific issues or technical areas, and produce independent recommendations for deliberation at the World Health Assembly. Two examples are the Consultative Expert Group on Research and Development: Financing and Coordination (CEWG)^28^ and the International Health Regulations Review Committees.^29^

## 2 Methods

The COnsolidated criteria for REporting Qualitative research (COREQ)^30^ were used to report on the characteristics of the research team, study design, and data analysis (Additional file S1, Supporting Information). COREQ was originally developed to promote explicit and comprehensive reporting of qualitative health research involving the use of interviews and/or focus groups to explore preferences and needs of clinicians, healthcare providers, policymakers, and patients. Its relevance goes beyond healthcare research since COREQ's items promotes transparency and clarifies choices made when designing the study, and collecting and interpreting the qualitative data.

### 2.1 Definitions

This study defined “SAC” in a broad way: a group of individuals with some kind of expertise that provide advice to decision‐makers informed by evidence from the natural and social sciences. “Scientific advice” includes assessments, guidelines, recommendations, and other types of prescriptions. “Design features” were defined as those features of the process, composition, and outputs of a SAC that are amenable to change by those convening these bodies.

### 2.2 Theoretical Perspectives

Insights from the scholarly literature exploring three interrelated concepts and frameworks for thinking about SACs come together to form the theoretical underpinnings of this study. The first is the extensive literature that has explored the characteristics of science–policy interfaces (SPIs). Two different ways of thinking about SPIs are particularly useful for this study. First, van den Hove's definition describes SPIs as “social processes which encompass relations between scientists and other actors in the policy process, and which allow for exchanges, coevolution, and joint construction of knowledge with the aim of enriching decision‐making.”^31^ This definition recognizes that translating scientific knowledge into helpful recommendations for policymakers is a process that goes beyond evaluating the quality of the scientific knowledge, and necessitates a joint process involving policymakers and other stakeholders. A second useful way of thinking about SPIs is offered by Koetz et al., who have defined SPIs as “institutional arrangement that reflect cognitive models and provide normative structures, rights, rules, and procedures that define and enable the social practice of linking scientific and policymaking processes” and which “assign roles to scientists, policymakers, other relevant stakeholders and knowledge holders and help guide their interactions according to specific principles and purposes.”^32^ Both definitions recognize that the *design* of SPIs have implications for how different stakeholders interact and how scientific advice is brought to bear on decision‐making processes. Both definitions depart from the much contested linear model for the relationship between science and policy, which assumes a unidirectional relationship where the mere existence of scientific studies and assessments—and sufficient level of interaction between scientists and policymakers—is expected to drive corresponding policy changes.^33^ The shortcomings of the linear model have been widely discussed and debated elsewhere.^34^, ^35^, ^36^, ^37^ Instead, the prevailing definitions of SPIs emphasize a collaborative model for thinking about how scientific advice can be integrated into decision‐making.^32^ Here, scientists and decision‐makers are expected to “negotiate what information is needed, what evidence is acceptable for the policy process and what the policy options are”.^11^ In this study's context, the prevailing definitions of SPIs are useful for understanding the interaction among scientists and different users of scientific advice (e.g., WHO staff, policymakers, civil society leaders) during the development of scientific advice. These definitions are also helpful for considering the various factors that must be addressed when structuring SPIs to maintain scientific independence and integrity. This is particularly crucial to WHO, since at the heart of WHO's normative authority lies the ability to structure independent and evidence‐informed processes to produce credible scientific assessments on health issues. In WHO, the principles and methodology guiding the agency's production of evidence‐informed recommendations have in part evolved with reforms of its guideline development process (see more below under “Study setting, context, and scope”), with some of its guideline development panels more recently implementing models for considering research evidence together with information on other factors (e.g., values and preferences, resource implications, feasibility) in a structured and transparent way when producing evidence‐informed recommendations.^17^, ^19^, ^38^

Boundary organization is a second useful theoretical concept, describing organizations that manage SPIs.^39^, ^40^ An extensive review of the theoretical development and empirical application of this concept exists elsewhere.^40^ A related term is that of “boundary spanning,” which refers to the “work to enable exchange between the production and use of knowledge to support evidence‐informed decision‐making in a specific context,”^41^ and where boundary spanners are individuals or organizations that actively facilitate this process. Boundary organizations and boundary spanners are concepts that were developed separately at different times,^39^, ^42^ and are considered to be distinct from one another.^39^ However, in the context of this study, we consider both concepts to describe how actors like WHO staff facilitate processes by which scientists assess and reach consensus on the scientific knowledge and present these to decision‐makers, and how they facilitate processes by which decision‐makers consider and accept (or do not accept) the scientific knowledge as accurate and authoritative. Finally, this study drew on a theoretical framework initially developed by Cash et al. that suggests quality, relevance, and legitimacy as the three key determinants of the effectiveness of scientific advice.^7^, ^8^ This framework is well established in the literature on scientific assessments in global environmental governance.^2^, ^3^, ^5^, ^9^, ^10^, ^11^, ^43^, ^44^ It was developed by reviewing the research exploring the interaction between science and decision‐making, consulting with scientists, practitioners, and decision‐makers, and comparing four cases of scientific advisory systems on different issues in different settings. Cash et al. define “credibility” (quality) as the “scientific adequacy of the technical evidence and arguments;” “salience” (relevance) as the extent to which the scientific advice is responsive to the particular concerns and needs of decision‐makers; and “legitimacy” as the extent to which the process of generating the advice has been “respectful of stakeholders divergent values, unbiased in its conduct, and fair in its treatment of opposing views and interests.”^7^ A key assumption underpinning the pursuit of quality, relevance, and legitimacy is that these are interdependent attributes. This means that individual design features may actually enhance more than one attribute at a time or may bolster one attribute at the expense of another. The interview guide for this study was designed to elicit WHO staff's general views and perceptions of design features that are important for quality, relevance, and legitimacy as well as specific design features perceived to be more tightly linked to the attributes with the aim of capturing choices that could force trade‐offs among these attributes.

Previous studies applying this framework have primarily focused on how the institutional design of SACs might increase *perceived* quality, relevance, and legitimacy of the advice. In the context of this study, we were also interested identifying key design features that could become standards for quality, relevance, and legitimacy that are independent of the immediate perception of external stakeholders. In other words, this study was interested in drawing from WHO staff's experience with designing SACs to identify if there are design features that have so tight a relationship with one or more of quality, relevance, and legitimacy, that their presence could become metrics for the de facto quality, relevance, and legitimacy of the advice. That said, this study did not assume that all stakeholders would identify relationships among different design features and quality, relevance, and legitimacy in exactly the same way, nor did it aim to propose a standardized way of assessing the quality, relevance, and legitimacy of SACs' advice on the basis of WHO staff's experience. Moreover, this study also expected that some interviewees would emphasize how different design features engender recipients' *perceptions* of quality, relevance, and legitimacy—consistent with other studies investigating these questions.^3^, ^8^, ^9^, ^11^

Accordingly, we applied a modified form of Cash et al.'s framework. First, “quality” was used instead of “credibility”. WHO has in a previous assessment of their mechanisms for securing external expert advice focused on how to improve the efficiency, legitimacy, and quality of their expert advisory bodies and their products.^45^ Specifically, among the features considered to contribute to the quality of scientific advice include the process of its development, use of evidence, and peer‐review— consistent with a belief that there exists some standards by which the quality of the scientific advice can be assessed. The second modification of the framework pertained to replacing the word “salience” with “relevance.” Both terms have been used interchangeably in the academic literature;^2^, ^9^, ^11^, ^43^ we chose “relevance” thinking that this term would be more easily understood in WHO's context.

### 2.3 Study Setting, Context, and Scope

WHO is the “directing and coordinating authority for international health work” within the UN system.^12^ Its headquarters are in Geneva, Switzerland. All interviewees in this study were WHO staff working at headquarters. In the context of WHO, the terms used more commonly instead of scientific advice are “guidelines,” “norms,” and “standards.” A more general term used by WHO for documents that have normative content is “normative instruments.”^46^ A recent evaluation of WHO's normative function^26^ distinguished between two broad groups of normative instruments: 1) conventions, regulations, and recommendations endorsed by the World Health Assembly or adopted by an equivalent body; and 2) a broad range of normative guidelines prepared by WHO's staff, including the SACs established by them. This study is primarily concerned with the use of SACs to develop the latter group of normative instruments. However, normative instruments developed by SACs often inform normative instruments that are endorsed by the World Health Assembly. Different types of SACs are convened and managed by WHO to generate scientific advice, and the target audience for these bodies can differ greatly. The outputs from WHO's guideline development processes and expert committees, for example, are generally not discussed by the World Health Assembly, but rather published and disseminated directly to reach health policymakers, health system managers, and health professionals. In comparison, reports and recommendations from committees have fed directly into decision‐making processes of member states. It is also worth distinguishing between two groups of decision‐makers: WHO staff and WHO member states. Most of WHO's SACs direct their advice to WHO, and the agency is then responsible for forwarding their recommendations to WHO member states. However, recommendations from high‐level commissions, special committees, and expert working groups are more commonly disseminated directly to member states.

WHO's processes for developing scientific advice have in the past faced intense scrutiny. In 2007, a study by Oxman et al. identified major flaws in WHO's guideline development processes, including a lack of systematic and transparent methods for retrieving, appraising, synthesizing, and interpreting evidence, and rarely including methodologists or representatives of populations affected by the recommendations when developing guidelines.^47^ In response to this study and the criticism that followed, WHO implemented several institutional changes, including establishing a Guidelines Review Committee to independently review all guideline proposals from WHO's departments and to approve the final guidelines once completed. WHO also committed to prioritizing the use of systematic reviews to inform its guidelines and to use the Grading of Recommendations Assessment, Development, and Evaluation (GRADE) tool to evaluate the quality of included evidence and assist in making judgments about the strength of recommendations.^48^ This study is limited to investigating and drawing insights from the general experience across WHO's different SACs, rather than the specifics of WHO's guideline development process. Scrutiny of WHO's SACs was further instigated by criticism from the British Medical Journal in 2010 about the lack of transparent processes and public disclosures by WHO of declarable interests among experts in the Emergency Committee established to advise WHO about the declaration of the influenza A(H1N1) pandemic in 2009.^49^, ^50^ In response, former WHO Director‐General Margaret Chan explained that keeping the names of the emergency committee private “was motivated by a desire to protect the experts from commercial or other influences” and that “at no time, not for one second, did commercial interests enter my decision‐making,” while acknowledging that “WHO needs to establish, and enforce, stricter rules of engagement with industry, and we are doing so”.^51^ The International Health Regulations Review Committee that evaluated WHO's performance during the influenza pandemic reported “no evidence of attempted or actual influence by commercial interests on advice or decisions taken by the WHO,” but also that “WHO should clarify its standards and adopt more transparent procedures for the appointment of members of expert committees, such as an Emergency Committee, with respect to potential conflicts of interest,” including disclosing the identity and relevant background, experience, and relationships of Emergency Committee members at the time of their proposed appointment.^29^ The selection of members and the decision‐making processes for adding medicines to WHO's Model List of Essential Medicines have also undergone similar debate.^52^, ^53^, ^54^, ^55^ This study thus benefits from learning about SACs within WHO that have been scrutinized, criticized, and changed over time to improve their institutional designs and processes.

### 2.4 Document Review

To identify what is considered “good practice” within WHO for convening SACs, a review was conducted of existing guidance in the agency on how to convene and manage different types of SACs. This review was used to inform and draw comparisons to insights from the interviews. Documents reviewed included WHO's Handbook on Guideline Development,^16^ the regulations of expert advisory panels and committees described in WHO's Basic Documents,^14^ and a sample of terms of references of scientific and technical advisory groups (STAGs) collected from WHO's website.^56^, ^57^, ^58^, ^59^, ^60^, ^61^, ^62^, ^63^, ^64^, ^65^, ^66^, ^67^, ^68^ These documents were used to identify guiding principles for designing SACs. These guiding principles were divided into three categories that broadly reflect three key aspects of SACs: 1) principles guiding the process of creating SACs; 2) principles guiding the selection of SAC members; and 3) principles guiding the processes by which outputs of SACs are produced and disseminated.

#### 2.5 Primary Data Collection: Semistructured Interviews

#### 2.5.1 Interview Guide

A semistructured interview guide of 14 questions (**Table** **2**) was developed to draw lessons from the successes and challenges that WHO has faced in the design and effectiveness of its SACs. The interview guide included questions about: the interviewees' experience with convening SACs; design features interviewees deemed to be important for quality, relevance, and legitimacy of a SAC's advice; dimensions of diversity the interviewees deemed to be important; and strategies for safeguarding scientific independence. All interviews followed a semistructured format, permitting some adaptation of the questions depending on interviewees' responses, and flexibility to probe more deeply into unexpected but interesting themes that emerged.

**Table 2:** Key informant interview guide

**Background information** I will ask questions about (a) your organization's SACs, (b) what makes SACs work effectively, (c) your ideas for improving SACs, and (d) some final questions. Interviews will be recorded, transcribed, and anonymized, but participants can still choose to go off‐record at any time, up until publication of results. You can of course always ask any questions of the research team at any time. In the context of this project, we are defining SACs as: “(a) a group of individuals with some kind of expertise (b) that provides advice to internal or external decision‐makers (c) based on evidence from the natural or social sciences.” This would include most entities called “scientific advisory committees,” “expert committees,” “study groups,” “review panels,” “commissions,” etc., but not “research ethics boards” or “governing bodies.” **Questions** 1. What is your role in convening SACs at your organization? 2. Under what circumstances does your unit convene a SAC? 3. Are there best practices at your organization for guiding how you convene a SAC? 4. From your experience, what are important design features of SACs that contribute to their effectiveness? 5. Of those design features you identified, which is the single most important design feature for ensuring a SAC's advice is: a) high quality?; b) relevant?; and c) legitimate? 6. In what way might the management of SAC be affected if the issue addressed is: a) scientifically complex?; and b) politically controversial? 7. What dimension of diversity is most important when selecting SAC members? What other dimensions of diversity are extremely important? 8. What is the optimal size of a SAC? 9. What specific steps should be taken to safeguard the scientific independence of SACs? 10. What steps do you take to ensure effective dissemination and uptake of SAC's advice by the targeted stakeholders? 11. Where is the greatest potential for improving how SACs are designed at your organization? 12. If you had unlimited resources to convene SACs at your organization, what improvements would you then make? 13. In your opinion, when are SACs underutilized and when are they over‐utilized at your organization? 14. Is there anything else you would like to add about this subject which I haven't asked you about?

#### 2.5.2 Recruitment and Selection

At the time of the interviews, staff at WHO headquarters were organized into seven main clusters, of which two were leadership and management (i.e., Director‐General's Office and General Management) and five were devoted to broad health themes: Family, Women's and Children's Health; Health Systems and Innovation; HIV/AIDS, TB, Malaria, and Neglected Tropical Diseases; and Noncommunicable Diseases and Mental Health (NMH). At the time of writing, the Director‐General and WHO's new management is restructuring and renaming the various clusters and departments. The organigram included with this article (Appendix 1) reflects WHO's structure at the time when the interviews were conducted, with the exception of the Health Emergencies Programme that was established later in October 2016.

Each cluster is divided into departments that deal with specific health issues, such as maternal, newborn, child, and adolescent health; reproductive health and research; or HIV/AIDS. An initial email introduction to the directors of these departments was made on behalf of the study team by a very senior leader within the agency, which introduced potential participants to the study team, outlined the objectives of the effort, and encouraged participation. A final list of 68 potential WHO interviewees was generated through a combination of purposive and snowball sampling. From this sampling frame, 41 WHO staff accepted an invitation to be interviewed. The remaining 27 WHO staff either forwarded the invitation to one of the other interviewees in the sampling frame, declined participation (generally reporting lack of time), or did not respond.

#### 2.5.3 Interviews

A total of 41 interviews were conducted between March and June 2016. Six of these were pilot interviews conducted in‐person by SJH (JD PhD, male researcher) at WHO headquarters in Geneva to assess the relevance of the study, identify some preliminary hypotheses, and fine‐tune interview questions to maximize their clarity and probative value.

These interviews were not audio recorded and transcribed for further analysis. The remaining 35 interviews were conducted by UG (MD PhD, male researcher), and audio recorded, transcribed, and anonymized. Every participant was sent an email prior to the interview briefly introducing the interviewer and attaching a summary of the study. Thirteen of these 35 interviews were conducted in‐person at WHO's headquarters in Geneva; the remaining 22 interviews were conducted by telephone. Three interviewees from the same department were interviewed together. Nonparticipants of the study were not present during the interviews, and no repeat interviews were carried out. The interviews lasted between 45 and 60 minutes. We observed that the last 5‐6 interviews only introduced a few new codes and no new major themes, which we used as the indication for reaching data saturation. Transcripts and a 1–2 page summary of the interview were sent to every participant for feedback approximately one year after the interviews were conducted, upon which five interviewees returned minor feedback. The final manuscript was sent to all interviewees; four participants provided feedback. All authors have previously designed, conducted, and published studies involving qualitative methods.^69^, ^70^, ^71^, ^72^

### 2.6 Qualitative Data Analysis

The qualitative data from the interviews were organized and interpreted using an inductive approach involving the general five‐cycle strategy described by Yin.^73^ The five main steps of this strategy are 1) compiling, 2) disassembling, 3) reassembling, 4) interpreting, and 5) concluding. *Compiling* entailed transcribing and further deidentifying each audio file, including removing specific mentioning of positions, departments, or persons that may indirectly identify the interviewees. *Disassembling* consisted of open coding where codes, including in vivo codes, were assigned to words, phrases, and larger pieces of each interview transcript. During this stage of the analysis, we drew on the theoretical perspectives informing this study, and in vivo codes describing design features were categorized according to whether interviewees considered these to affect quality, relevance, and legitimacy of SACs' advice. Similarities between codes from different interviews were identified and defined as categories, thereby facilitating an incremental understanding of the data towards a higher conceptual level. Further, a series of hand‐written memos describing the investigators' thoughts about the interviews, concepts, and ideas that emerged during the initial phase were documented to help compare whether later interpretation of the data were similar to or departed significantly from the initial impressions. The *reassembling* phase consisted of bringing codes and categories together in order to identify broader patterns in the data. The codes were continuously processed following grounded theory's constant comparison method in order to identify major themes describing interviewees experience with convening and managing SACs. Coding during compiling, disassembling, and reassembling was conducted by the lead author (UG), and the major themes emerging from codes and categories were discussed, interpreted, and agreed with the co‐authors (SJH/TO). The raw file of major themes and underlying codes and categories are available in Additional file S2 (Supporting Information).

The identification of negative cases and rival thinking were used as strategies to minimize the risk of investigator bias. During the *interpreting* phase, the reassembled data were used to write a narrative around the study aims, while continuously assessing whether revisiting the disassembling and reassembling phases were needed to recompile the data. During the *concluding* phase, the main empirical findings were interpreted in light of the broader research literature that address issues similar to the ones raised by this study. Throughout this five‐cycle analytic strategy, there was recursive movement between the phases in order to continuously reconsider the codes and interpretation of themes, and to overall optimize insights from the data.

### 2.7 Ethics

Ethics exemption was granted by the University of Ottawa's Office for Research Ethics since this study was considered to be a program evaluation, in accordance with Canada's Tri‐Council Policy Statement on Ethical Conduct for Research Involving Humans (TCPS2).^74^

## 3 Findings

### 3.1 SACs are Primarily Created to Respond to Technical Needs But Can Also Serve Broader Strategic Objectives

Interviewees described that the primary motivation for convening SACs was to deliver on WHO's normative functions and respond to member states' technical needs by producing credible scientific assessments on health issues. SACs were considered instrumental for acquiring expertise not available within the agency and for obtaining independent scientific advice. For example, interviewees expressed that the agency would “decide to establish a committee when we believe there is a topic at stake that requires more in‐depth analysis, more in‐depth assessment, in‐depth recommendations” and that the need for this would be identified by talking to countries and other partners, and understanding “what's not been addressed in a serious way” (WHO interviewee 22).

Yet meeting technical needs was not the only motivation for establishing SACs. Interviewees expressed that SACs were strategically important to WHO for several reasons. First, SACs were seen as valuable bodies for facilitating broad participation and ownership of scientific advice. Second, SACs were seen as instrumental for strengthening the visibility of an issue. Third, SACs were considered a crucial instrument for following an evidence‐based process to act on politically controversial issues. For example, one interviewee expressed:

“they (member states) are encouraging us to generate the best evidence, because quite often, in politically controversial areas, evidence is fairly weak. So our role is in controversial areas, to ensure the evidence is as strong as possible” (WHO Interviewee 27). Moreover, interviewees expressed that SACs could be valuable “testing grounds” for promoting ideas and recommendations that WHO not yet is prepared to promote on its own. For example, two interviewees expressed:

“So, sometimes, a scientific advisory committee may be convened to ensure political cover for the organization, rather than to provide much‐needed technical input. So, in that instance they may be overutilized.” (WHO Interviewee 5).

“if there are things there that could be controversial, and we don't want WHO to be implicated yet because there's no political will or political readiness to embrace something that the experts have said, we just say in a caveat, in the technical report series, that these are the opinions of the experts, and not necessarily of the organization. In that way we don't stifle their expertise, but at the same time we protect the organization. Because it's true, there are times that our experts have said a lot things that we're not yet ready to embrace. And that's fine. But we just need to make sure that some member states who might attack us for those opinions, we just state it out there that these are the opinions of the experts, these are not the opinions of WHO.” (WHO Interviewee 34).

A recent evaluation of WHO's normative function reinforce the observation that SACs can serve broader strategic objectives. It noted that “preparing strategies and guidelines has to some extent become a way for attracting attention and recognition to a certain area of work,” and suggested that normative products functioned as an advocacy tool to gain internal recognition, and raise awareness globally on neglected issues.^46^ Moreover, specific examples of the way WHO uses its various SACs reinforces the point that the primary purpose vary between producing credible scientific assessments and serving broader strategic objectives. Commissions tend to be established to produce high‐level political messages and elevate priority for global health issues on the global political agenda. On the other hand, WHO's guideline development process and its advisory panels and expert committees can be considered primary instruments for delivering credible scientific assessments through systematic, structured, and transparent processes. These instruments have previously been under intense scrutiny for perceived lack of transparency of the decision‐making process.^47^, ^52^, ^75^ Reflecting on the different types of SACs that exist in WHO, one interviewee expressed the need for external stakeholders to better understand the differences across them and the nature of their scientific advice:

“we have different hierarchy of committees. The ones that are mandated by the WHA, where the Director‐General appoints commissioners or advisory board members or whatever, or those where the technical departments just want an outside view. Two different things, both very important, but of totally different nature. Therefore, the legitimacy also is dependent on the understanding of what level we're talking about. It is also important that both members of such a committee and the outside world are clearly aware of the different nature of these committees that we have in the WHO” (WHO Interviewee 8).

### 3.2 Independent SACs Require Autonomy from the Institutions Convening the SAC, from the Institutions Where SAC Members Work, and from the Institutions to Whom the Advice is Directed

WHO interviewees described a range of strategies for assuring the independence of scientific advice. Particular emphasis was placed on three key aspects: 1) autonomy from the institution convening the SAC (in this case, WHO); 2) autonomy from the institutions where experts work; and 3) autonomy from institutions to whom the advice is directed.

With respect to maintaining autonomy from WHO, one positive aspect raised about current practice was that experts do not receive remuneration, and that this was important for maintaining the experts' independence:

“We also don't want our members to be paid for what they are doing. I think it is important that the members of the committee not be paid and they don't do contract‐like work for us…then we think there will be a conflict of interest situation” (WHO interviewee 12).

The second aspect was that the experts should be autonomous of the institutions from where they are recruited and the governments of their own countries. Several interviewees expressed that many experts had to be educated about their role, and the need to act and express themselves in their personal capacity:

“and that's something else that we have to remind people significantly about, that they're not there representing their government or their institution” (WHO interviewee 16).

Moreover, interviewees described that strategies for managing conflicts of interests primarily focused on financial interests, and that current practice gave insufficient attention to managing institutional and other non‐financial interests. For example, one WHO interviewee expressed:

“we don't deal with the institution, so if you come from an NGO that is engaged in certain kinds of projects, does that change your ability to look at questions in an objective fashion?” (WHO interviewee 9).

Finally, interviewees underlined the importance of SACs being autonomous of institutions to whom advice is ultimately directed, including governments and philanthropic organizations. Several interviewees described how WHO's increasing dependence on voluntary contributions is becoming an emerging problem with respect to ensuring complete independence of its SACs. It was expressed that in some areas conflicting interests may emerge from voluntary contributions to fund scientific advisory processes, for example when a donor “might also be the funder of some trial that is going on, for example, and they are not supposed to fund the guideline development process” (WHO interviewee 17).

### 3.3 Designing SACs is an Exercise of Balancing Trade‐offs Among Quality, Relevance, and Legitimacy

Existing guidance within the agency about how to design and manage SACs were reviewed to identify key principles that currently guide decisions related to the creation, composition, and outputs of SACs. Moreover, interviewees were asked questions about which design features they deemed the most important for ensuring the quality, relevance, and legitimacy of the advice. The insights from the reviewed documentation and the interviews were combined to identify key principles that are considered to be “good practice” within WHO for convening SACs. We identified these principles to guide decisions related to three key aspects of SACs: the establishment of SACs; the composition of SACs; and the processes by which outputs of SACs are produced and disseminated (**Table** **3**).

**Table 3:** Key principles guiding the design of WHO's SACs and perceived primary relationships with quality, relevance, and legitimacy.a)

	Quality	Relevance	Legitimacy
**Principles guiding the establishment of SACs**			
WHO acts as the secretariat to support and facilitate the work of its SACs, but the SAC should be independent of WHO		X	
Clarity is needed about the need, scope, and targets of the advice, including identifying: the format of the advice; who is likely to implement the advice; and what infrastructure and services are needed for implementation	X	X	
Recruitment should balance feasibility with transparency and comprehensiveness, and consider an open call for nomination where possible, in addition to drawing members from established technical networks and collaborating centers	X	X	X
Experts should not receive remuneration for participation, but should be compensated for their reasonable expenses			X
Experts should submit a declaration of interests form, which must be updated before each meeting and made publicly available	X		X
**Principles guiding the composition of SACs**			
Experts should be independent, serve in their personal capacity, refrain from promoting policies and views of their institutions, and not accept instructions from governments nor from other authorities external to WHO	X		
Balanced geographic representation and gender balance should be sought	X	X	X
A broad range of relevant disciplines, and different schools of thought, approaches, and practical experience from various parts of the world should be represented	X	X	X
End‐users of the advice should be represented where possible, including those who will adopt, adapt, and implement the advice		X	X
Communities and/or population groups most affected by the advice should be represented where relevant and possible		X	X
Funders of SACs may observe meetings, but should neither play any role in contributing to the appraisal of evidence informing the advice nor be involved in the formulation of the advice	X		X
Staff from other UN agencies are not eligible to serve on WHO's SACs, but may participate as observers			X
**Principles guiding the processes by which outputs of SACs are produced and disseminated**			
Decision‐making rules should be defined and made explicit before recommendations are formulated, including a plan for how to proceed if consensus cannot be achieved	X		
The selected chairperson should have general knowledge of the topic and experience engaging with consensus‐based processes involving people with different opinions, but not hold strong views about the issues and advice that is being considered	X		
Detailed preparation in advance of meetings	X	X	
Experts are not allowed to participate in deliberations on topics where they have a conflict of interest	X		X
Broad ownership of the questions explored and inclusive participation should be fostered		X	X
Smaller working groups of SAC members may be established to address specific questions	X	X	
Broad consultation process should be implemented		X	X
The process for developing advice should be explicit and transparent so that users see how and why a recommendation was developed, by whom, and on what basis	X		X
Divergent views about the evidence base and recommendations should be recorded, with the reasons for these diverging opinions explained			X
The text of reports and recommendations from SACs should not be modified without the consent of the SACs' members	X		X
Evidence used to inform advice should be made publicly available as fast as possible			X

^a)^ The perceived primary relationships are indicated based on insights from the interviews.

The interviews reinforced a key theoretical assumption in this study, namely the interdependent nature of quality, relevance, and legitimacy, where efforts to strengthen one of these attributes may bolster or weaken the others.^2^, ^7^ Interviewees raised several examples of the difficulties with striking the right balance across different dimensions when designing SACs, with implicit choices involving trade‐offs among quality, relevance, and legitimacy (**Table** **4**).

**Table 4:** Examples of design features demanding careful considerations of trade‐offs among quality, relevance, and legitimacy

Features	Considerations for quality, relevance and/or legitimacy	Illustrative quotes
Meeting tight timelines by recruiting experts with well‐known reputation more easily and rapidly through pre‐existing networks versus dedicating resources to manage an open call for nominations	Securing the preferred expertise can increase the quality of the advice, but recruiting from too narrowly confined networks could negatively affect diverse representation and risk bringing experts sharing very similar perspectives to the table, thereby risking diminishing the relevance and legitimacy of the advice	“there should be some kind of transparent process to make sure we get best sorts of people on these committee, and some kind of process which is nomination rather than just inviting people” (WHO interviewee 1) “I saw this in many areas, where people had a club of people they relied on to come to meetings, and that lead to a single view” (WHO interviewee 16) “if it is left up to one or a couple of people, it tends to be people they know, the networks they know, and really like‐minded participants” (WHO interviewee 20)
Securing an appropriate mix between experts recruited from reputable academic institutions and experts recruited more broadly to enhance the geographic representation in SACs	Well‐known experts from reputable institutions can strengthen the quality of the advice, but loss of geographic representation risks compromising relevance and legitimacy	“you need to take tough decisions on what are the diversity dimensions that are more important for you in this specific committee than perhaps in others” (WHO interviewee 8) “We have also seen that so‐called experts from Northern or developed countries may not even have the slightest idea of working under the conditions that our guidelines are telling them to” (WHO interviewee 14) “a lot of the expertise in the area in which we are mainly working is quite geo‐concentrated. A lot of the expertise actually, at least in the topics we discuss, is in the UK, the US, the Netherlands, and that's where a lot of the real experts, if you like, are. But obviously you can't just have meetings with people from those parts of the world” (WHO interviewee 16) “We try as much as we can to have geographic representation, but we won't compromise science and competency for a better scientific representation” (WHO interviewee 18) “we cannot have a meeting dominated by Americans and Europeans, which tend sometimes to be the case because many experts of course are in the best universities in the world, which happens to be in North America or Europe.” (WHO interviewee 22) “We need to constantly be trying to see how we cannot only, as an institution, we need to take some more proactive measures to get more engagement by the country on doing some of the scientific work. Unless we do it, our scientific advisory bodies will always be skewed” (WHO 34)
Securing an appropriate mix between experts from academia and experts working at an operational level	An overemphasis on recruiting academics to SACs could compromise the practical relevance of guidance	“We need to make sure the type of people convened on advisory committees are not just academia, we need a range of stakeholders, people that work obviously on the research, the primary research around themes, but also people that are implementers, that work on an operational level, that can give information and provide valuable aspects on how guidance could work or not work in such situations” (WHO interviewee 2) “I think it is equally important to have a balance between the technical experts and the doers, so between academics who are very well published on a topic, and people who have more operational and management experience of the same thing” (WHO interviewee 6)
Securing an appropriate mix between maintaining a fully transparent process and enabling a closed space where experts can discuss more freely without interference	Transparency is vital for SAC's legitimacy, but allowing space for closed discussions is necessary to strengthen the quality of the experts' discussions	“As you want a committee to deliberate freely, you also need to give them some space for doing so….Otherwise, you will not have this out‐of‐the‐box thinking, because people would not dare to say innovative things, because [they'd think] ‘oh, it's already quoted in the media, we haven't even looked at the likely consequences of a certain idea'. Therefore, you need to give them space and confidence, and we need to have this confidence in people that they are doing the right work, but then they need to come up with it, and make it public once they've all agreed on a certain idea” (WHO interviewee 8) “We only allow participation of experts who will come and inform the debate…why do we remove the observers? Because otherwise the committees would be dominated by organizations, or the funders, or people that are coming as observers but are actually not observing, they are influencing the debate” (WHO interviewee 12)
Securing an appropriate mix between tight management of conflicts of interests versus eliminating experts in spite of these not having a direct relationship with commercial entities with an interest in the subject matter	Management of conflict of interest crucial for quality and legitimacy, but very stringent policies risks in some technical areas, where few suitable experts are available, to compromise the quality and relevance of the advice	“…once we start applying rigidly the rules of conflict of interest, then you are supposed to identify if you belong to whatever university, and then people start asking, hold a second, the company that developed this drug is giving money somehow to the university…then that is seen as perhaps a potential conflict of interest. If we are very very strict, you end up, and this is the concern we have now, with people that are completely out of the business” (WHO interviewee 22)

Geographic representation was a design feature of SACs that all interviewees rated highly with respect to its impact on quality, relevance, and legitimacy, and which interviewees expressed to have particularly tight links to the relevance and legitimacy of the scientific advice. However, interviewees provided several examples of how insufficient effort to recruit and select members could inadvertently compromise geographic representation, as well as gender balance and other important dimensions of diversity. For some areas, a trade‐off where technical expertise was prioritized over geographic representation was viewed to be unavoidable since “in very technical areas, you have very few experts around the world, and if you want to put geographic balance as a priority you may miss the science and the expertise that you need” (WHO interviewee 18). Another interviewee expressed that the demands on WHO staff to respond timely to the needs of its member states sometimes forced its staff to rely on their own professional networks rather than recruiting experts more broadly or through a more transparent process:

“the time that you have to find the right experts is not long….what are the natural reactions, so you actually have a network of experts you are working with, and then the same people come to mind, so you have the usual suspects. This is something that, and rightly so, the WHO has been criticized for. But we have to understand the administrative background for doing this” (WHO interviewee 8).

However, overall, interviewees stressed that recruitment and selection processes should be transparent and avoid recruiting from narrowly confined networks to achieve a balanced composition of SACs, and that there should not be any excuses for not achieving the appropriate balance:

“Not if you try hard enough. It is the same thing you see with guidelines normally, where people say you can't find people without conflicts of interest. No, you can, you just have to look harder. Sometimes it takes a little bit of a while, sometimes you may have to do a couple of iterations, but it is very doable if you try” (WHO interviewee 16).

Accordingly, interviewees proposed several strategies, such as maintaining and updating a broader roster of experts, and supporting capacity‐building in LMICs to strengthen representation of LMIC contributors over the long‐term. In addition to the examples of choices described in Table 4, two additional aspects of SACs involving trade‐offs were identified in the interviews. The first pertained to the role of WHO staff, while the second was about increasing the potential impact of the advice by involving stakeholders who have the power to act on recommendations. Choices affecting both these aspects are crucial for understanding trade‐offs among quality, relevance, and legitimacy of SACs when navigating the interface between science and policy, and are discussed separately in the next sections.

### 3.4 Staff Supporting SACs Must Balance Between Safeguarding SAC Decision‐Making from External Influence, and Serving as a Broker between the Experts and the External Environment

WHO staff play a crucial role in supporting SACs with respect to clarifying mandates, articulating expected outputs, preparing background documentation, organizing meetings, and reporting the scientific advice. Interviewees highlighted different aspects of WHO staff roles as particularly relevant for the legitimacy and relevance of SACs' advice. For legitimacy, it was argued that WHO staff should protect SACs from political interference. For example, one interviewee expressed that “the technical and scientific advisors need to have freedom to operate from a scientific, technical perspective, alone, without fear that there is political oversight, somebody breathing down their necks seeing if they are giving a politically correct opinion rather than a scientifically correct opinion” (WHO interviewee 4). Moreover, it was expressed that WHO staff in collaboration with the chair of the SAC typically play important roles in ensuring that deliberations are based on the best‐available evidence and free from political bias:

“It is incumbent on WHO, it is incumbent on the chair, whoever is running it. You keep coming back to that, you want to make a statement, what is it based on? Is it based on views, your belief, or is there a good science behind that?” (WHO interviewee 9).

Moreover, interviewees expressed that the role of WHO staff was to provide administrative support, and not in any way compromise the legitimacy of SACs by influencing their deliberations. For example, two WHO interviewees expressed:

“I've experienced the difficulty, the challenge to stay away, to keep this independence of the committee…you want to say ‘oh, but this needs to be like this and that needs to be like that', because we believe there'll be, we know how things should work. But to keep this off, and to say, well, I need to empower this independence of this committee, rather than imposing technical matters on this committee, just because I would like to see this being reflected in their recommendations and report, is a challenge” (WHO interviewee 8).

“To me this is very important. They have to deliberate in an independent manner. It is not the WHO telling them what they should do, it's the group” (WHO interviewee 15).

At the same time, interviewees also described the importance of WHO staff staying actively engaged, and in different ways supporting the SAC with considering the broader political context when forming evidence‐informed recommendations. Since many experts are not always aware of the political sensitivities of the issues addressed, WHO staff could enhance this understanding:

“we need to make sure the members fully understand the political aspects, and not just are technical experts in these areas, but are also familiar with, and are able to discuss and advise on the politics of the science of what we are doing, and how the science we generate can be used in the political process” (WHO interviewee 28).

Moreover, WHO staff could facilitate the process of systematically making judgments about the benefits and harms of a policy option together with other criteria (e.g., costs and resource use, feasibility, values, and preferences, ethical, legal and societal aspects) that are relevant to consider when forming evidence‐informed recommendations, and thereby support the SACs to be more responsive to the needs of policymakers and other target audiences:

“but if you're not reflecting sometimes what this recommendation then would mean for the implementation in WHO, then of course a committee could go absolutely off tray and off track, and that's also a challenge” (WHO interviewee 8).

“The experts will provide advice, then you will need to know how to use that advice, and it's not automatic. The advice is not resulting always in a recommendation. This is where you need to balance this scientific advice and other determinants, other conditions, other arguments…” (WHO interviewee 32).

Overall, an internal tension within WHO can be observed about the appropriate role of staff managing SACs. On the one hand, the appropriate role of WHO staff is considered to be that of a “neutral administrator”—responsive to the needs of the SAC and providing administrative support, but not otherwise overstepping its role in a way that would compromise the independence of SACs, or create a perception that independence might have been compromised.^76^ On the other hand, a more proactive role is often promoted, where WHO staff serve as brokers between the experts that produce the advice and the stakeholders who have the power to act on them. Under this broad interpretation, SACs are instrumental for the WHO Secretariat's problem‐solving capacity, since these processes contribute to defining the problems of the day, identifying standards, and otherwise setting the options available to policymakers.^77^ Accordingly, WHO staff might bring their own experience and understanding of political sensitivities to bear on SACs' deliberations to maximize the potential for influence and impact by ensuring that the advice is responsive to the needs of decision‐making processes of member states and other actors. Such a role—facilitating dialogue and fostering agreement among scientists, policymakers, and other stakeholders affected or responsible for implementing the advice—is consistent with understanding the WHO Secretariat's role as a boundary organization acting at the interface between science and policy, with an overall aim of supporting evidence‐informed decision‐making.

### 3.5 The Need to Involve Stakeholders with the Power to Act on Recommendations Must be Balanced against the Need to Protect the Independence and Integrity of the Scientific Process

There was general agreement that the independence of SACs relied on clearly separating the scientific process leading to evidence‐informed recommendations from WHO's political processes—whether this was at the national or intergovernmental level—where recommendations may be debated. Accordingly, one interviewee expressed:

“we will typically stay away from touching on issues that are exclusive prerogative of member states, decided in the context of their political‐decision making process, but rather refer to evidence and best practices on an aggregate and global level” (WHO interviewee 6).

Implicit in this view is that SACs are most effective when their processes have adhered strictly to high‐quality methodology for acquiring, assessing, adapting, and applying the evidence base for interventions and policies to address health issues, and communicated undistorted to member states who can consider the advice within their own decision‐making processes. At the same time, some interviewees argued that SACs should be designed to engage with stakeholders within this broader context, since implementation requires recommendations to be accepted and acted upon by political actors. One strategy was to incorporate stakeholders that may be targets of the advice—such as funders, representatives of member states, and civil society organizations—as observers to SACs so as to facilitate implementation by “linking the advisory group more directly to actors who might use that advise” (WHO interviewee 3). Another interviewee expressed:

“So, what is the political context in which technical advice is being given, and what therefore needs to be taken into account to reflect diversity of stakeholders. So, therefore, one‐size does not fit all, but to ask the question ‘who are the main stakeholders we would like to implement those recommendations, thus they need to be represented on such a committee'” (WHO 8).

Some interviewees called for even more proactive engagement and mechanisms to engage more closely with policymakers when crafting scientific advice:

“...all the parts of systematic reviews and all these things should be something that is very much driven, not by policymakers, but scientists and academics, but when you draft recommendations based on the outcomes, it is important to involve policymakers because the recommendations have to be science‐based, but they have to also be realistic.” (WHO 18).

Similarly, another interviewee underlined the importance of sensitizing recommendations to the political environment in which these will be debated. This interviewee used the report of the International Health Regulations Review Committee from 2011 as a concrete example of a report which was technically excellent, but failed to attract the political attention needed to be prioritized for implementation:

“This is a lesson we've all learned from the International Health Regulations, wonderful report in 2011, you read the report and you think they have been really profoundly right in their recommendations, from a technical side, excellent. However, as the recommendations had not been vetted, not been discussed with policymakers out there, who obviously had a different understanding, or who saw different bottlenecks for implementation, therefore not much has happened. It was not on their priority list, it was not on their radar screen. So, therefore, and they perhaps did not see the urgency of why this needed to happen” (WHO interviewee 8).

Attuning SACs to the broader political context by enabling scientists, policymakers, and other relevant stakeholders to link up and jointly develop knowledge to enrich decision‐making processes seem sensible to increase the impact of SACs. Yet this can also compromise the ability of scientists to work freely in accordance with stringent professional standards.^76^, ^78^ Such integration can therefore raise questions over the independence and integrity of the advice, and thereby come with the risk of compromising quality and legitimacy. Accordingly, those convening SACs in WHO are faced with the challenge of structuring the interaction with policymakers and others actors, who bring different interests and preferences to the table, in a way that does not compromise the independence and integrity of the scientific process.

## 4 Discussion

WHO has a long and rich experience with convening SACs to inform policy and practice. The organization has previously demonstrated willingness to reform the procedures of its SACs, such as with its guideline development process.^79^ Three aspects about the design of SACs are worth discussing further in light of the study findings. First, whether there is a need for clarifying the typology and primary purpose of SACs in WHO, and the kinds of questions the different SACs are designed (and not designed) to address. Second, the need for SACs to transparently report the different stages of its work to protect scientific independence and integrity, including all factors considered to inform judgements about recommendations. Finally, the need for WHO to embark on strategic efforts to strengthen diverse representation in its SACs to further enhance the quality, relevance, and legitimacy of its advice. Together, these three are areas WHO could consider to further bolster its system of SACs to engage more effectively at the interface between science and policy (**Table** **5**).

**Table 5:** Further bolstering WHO's system of SACs

1. Clarify typology: clarify the typology of its SACs, including clarifying the distinction between those SACs that are established primarily for consensus‐building to produce credible scientific assessments versus those established primarily for consensus‐building to promote high‐level political messages. 2. Transparent reporting: design SACs to transparently report the different stages of its work, including all factors considered to inform judgements about recommendations. 3. Strengthen diverse representation: embark on strategic efforts to strengthen diverse representation in its SACs over time, and thereby enhance the perceived quality, relevance, and legitimacy of their scientific advice

### 4.1 Clarifying the Typology and Primary Purpose of its SACs to Avoid External Confusion over the Nature of their Scientific Advice

At the heart of WHO's normative authority lies its ability to convene SACs, and produce scientific assessments and evidence‐informed recommendations on global health issues. For the quality, relevance, and legitimacy of WHO's scientific advice, the agency could benefit from even greater clarity on the purpose and processes of its various SACs, including their primary purpose, how well equipped these are to respond to different types of questions, and the type of advice these offer.

Instruments used by WHO to produce dietary guidance for the prevention and control of noncommunicable diseases (NCDs) provide illustrative cases for why such clarity can be helpful. In 2015, WHO released recommendations calling on adults and children to reduce their daily intake of free sugars to less than 10% of their total energy intake.^80^ Crucial to the quality and legitimacy of the recommendations was the standardized, transparent, and rigorous guideline development process involving the use of the GRADE approach for evaluating the quality of the evidence‐base and evaluation of the process by WHO's Guidelines Review Committee. This has likely helped WHO withstand push‐back from industry, as well as Member States promoting industry interests, both during the development of the guidance and after its release.^81^

Taxation of sugar‐sweetened beverages is another recommendation promoted by WHO in recent years.^82^ This recommendation has been subject to deliberation by three different SACs convened by WHO.^25^, ^83^, ^90^ None of these have been WHO's formal guideline development process, which in WHO's own words is necessary for a recommendation to be construed as a WHO‐endorsed recommendations.^84^ Still, the sugar tax has been picked up by media^85^, ^86^, ^87^ and among scholars^88^ as a WHO recommendation. The first time WHO came out strongly in favor of a tax on sugar‐sweetened beverages to prevent obesity was in conjunction with WHO's 2016 report on “Fiscal Policies for Diet and Prevention of Noncommunicable Diseases,” which was a result of a technical meeting facilitated by the WHO Secretariat and involving representatives from academia, policy, and civil society.^25^ An effective tax on sugar‐sweetened beverages was later recommended by a different type of SAC, namely the Commission on Ending Childhood Obesity.^83^ In contrast, the recent WHO Independent High‐level Commission on NCDs (NCD Commission), convened to advise the WHO Director‐General on bold recommendations on how countries can accelerate progress towards SDG target 3.4 on the prevention and treatment of NCDs and the promotion of mental health and wellbeing, omitted taxation of sugar‐sweetened beverages among its recommendations.^89^, ^90^ This omission received considerable media attention.^91^, ^92^, ^93^

The NCD Commission—composed of representatives of governments, UN agencies, NGOs, the private sector, philanthropic foundations, and academic institutions—was never designed to assess the evidence‐base for policy options and produce a credible scientific assessment. Instead, it was designed to be a consensus‐building process to promote high‐level political messages. In response to the NCD Commission's omission, the Director‐General of WHO was quick to publicly declare on Twitter that “taxing sugary drinks is an effective way to reduce sugar consumption and decrease the risk of diabetes & obesity” and that WHO's position stands firm.^94^ However, for external stakeholders who lack a nuanced understanding of the different type of SACs that exist in WHO, the NCD Commission's omission of sugar tax among its recommendations carries the risk of creating ambiguity about the evidence‐base for a tax on sugar‐sweetened beverages. This might especially be the case for two main reasons: 1) the NCD Commission itself promoted the message “There is no excuse for inaction, as we have evidence‐based solutions,”^90^ and by promoting the recommendations it endorsed as evidence‐based, it indirectly calls into question the evidence‐base for policy options that were excluded, such as the sugar tax; and 2) WHO has actually never subjected its advice on sugar tax to the most structured, transparent, and rigorous process it has for producing high‐quality, relevant, and legitimate evidence‐informed recommendations—namely its formal guideline development process.^16^

Overall, clarifying the typology and primary purpose of WHO's SACs, and the kinds of questions the different instruments are designed (and not designed) to address might help promote more prudent use of SACs, and over time help further foster the quality, relevance, and legitimacy of the scientific advice from WHO's various instruments.

### 4.2 SACs Should be Designed to Transparently Report the Different Stages of Its Work to Protect the Independence and Integrity of the Scientific Process

Increasing the relevance of scientific advice by considering the needs and preferences of policymakers and other users can be pivotal for impact. To seek increased relevance, conveners of SACs might consider interacting more closely with policymakers and other actors, for example by involving them as members of SACs or enabling other forms of close interaction during the process of developing scientific advice. This might especially be the case when SACs are established primarily to promote high‐level political messages. In these cases, it is imperative for the perceived quality and legitimacy of the scientific advice that the SACs are designed in a way that do not impair the mechanisms by which evidence is appraised and synthesized, or impairing the processes in such a way that actors guided by ideological or commercial interests can question the integrity of the experts involved. To deal with this challenge, conveners of SACs should consider transparently dividing its work into different stages, and report on the contributions of different stakeholders (including the Secretariat) to these stages, including how these inputs informed the process of developing scientific advice. What is imperative for the legitimacy of SACs is clearly separating the process by which SACs appraise, synthesize, and make judgments on evidence from the processes and inputs used to contextualize the scientific advice to be responsive to policymakers. In another article in this collection, Rosenbaum and colleagues present the Evidence to Decision (EtD) framework to support the process of moving from evidence to decisions.^38^ The EtD separates between three aspects of developing scientific advice: (1) the judgment—the option chosen by the SAC; 2) the research evidence collected in a preplanned and rigorous fashion to inform a judgment and 3); additional considerations to inform or justify each judgment. The latter may include practical experience, concerns over feasibility, and other considerations that contribute to contextualizing the advice. Crucial for the legitimacy of SACs is to be transparent about the content of these additional considerations and how these have been used during the process of developing scientific advice. Frameworks such as EtD for systematically and transparently reporting the process from evidence to decisions can support SACs to consider inputs from stakeholders in the external environment while protecting the quality and legitimacy of the scientific assessment.

### 4.3 Strategic Efforts to Strengthen Diverse Representation in WHO's SACs can Contribute to Strengthening Quality, Relevance, and Legitimacy of Their Scientific Advice

In the intergovernmental context, trade‐offs that sacrifice diverse representation reduce the perceived relevance and legitimacy of scientific advice, thereby diminishing the ability of SACs to contribute helpfully to decision‐making processes. For example, several scholars have shed light on the lack of trust among many low‐ and middle‐income countries in the assessments from the Intergovernmental Panel on Climate Change since few scientists and policymakers from these countries had participated.^95^, ^96^ In van de Hel and Biermann's recent study of six science institutions in global environmental governance, they identified that representation of relevant scientific expertise, geographic representation, and gender balance are the main factors by which SACs claim legitimacy.^3^ Yet they found that “achieving the objective of disciplinary, geographical and gender balance often proves difficult in practice.”^96^

Securing diverse representation in SACs has been a persistent and long‐term challenge in the UN system.^76^ A partial explanation is the uneven distribution of scientific capacity among regions. Moreover, as indicated in the interviews of this study, lack of awareness, effective procedures, and effort to identify scientific expertise across regions are also contributing factors. This suggests the need for WHO to embark on strategic efforts and demonstrate a leadership role to strengthen diverse representation in its SACs over time. General strategies include continuously updating a roster of experts from across regions, and insofar possible implementing transparent and open calls. Specific actions include conducting trainings to strengthen the capacity among health scientists and implementers (e.g., frontline health workers, health system managers, public health professionals) in LMICs to participate in SACs. In those rare situations where WHO cannot identify the needed expertise from LMICs, reserving 2–3 seats for more inexperienced candidates for capacity‐building purposes could support efforts to strengthen diverse representation over time.

### 4.4 General Insights and Future Research

WHO's own experience from convening SACs provide some insights for the design of SACs more generally. Informed by the study findings, we propose five design features that all conveners of SACs should consider implementing to promote the quality, relevance, and legitimacy of scientific advice (**Table** **6**).

**Table 6:** Five design features that all conveners of SACs should consider implementing

Open call for nominations to enable transparent recruitment process and facilitate diverse representation (including geographic representation, gender balance, balance between experts from academia and experts with operational experience, involvement of representatives of communities affected by the advice) Procedures for disclosing any interests (financial, intellectual, institutional) with the potential to unduly influence the judgement of the experts involved, and for implementing appropriate measures for managing the reported conflict of interest Define and make explicit the decision‐making rules, and transparently separate the stages involving critical appraisal and synthesis of evidence from the stages where evidence is considered together with other types of inputs, including those from policymakers and other stakeholders among the targeted audience Transparently report on the inputs provided by different stakeholders, including the secretariat supporting the SAC, and how these inputs where considered when making judgments Provide explanations when the design and practice deviate from the above (e.g., for example when lack of resources or lack of time prohibits implementing open call for nominations, when urgent demand for evidence‐based advice requires deviating from standard systematic review procedures for searching, appraising, and synthesizing the evidence, or when diverse representation isn't achieved)

Going forward, sharing experiences with managing SACs across WHO, as well as learning from international institutions in other sectors, could facilitate the development of further guidance on how to design SACs to optimally balance quality, relevance, and legitimacy. Since WHO's SACs vary in design and mandate, more detailed studies of specific SACs in WHO's system should be commissioned to deepen our understanding of how effective these are in informing policy and practice. In particular, comprehensively studying the effectiveness of SACs requires examining the processes and pathways by which the scientific advice is taken up at the country‐level. By continuously evaluating the design of their own SACs, WHO can take a leadership role in their design and contribute to strengthening these institutions for the benefit of WHO, as well as the many governments and non‐state actors that rely on WHO for advice.

### 4.5 Strengths, Contributions, and Limitations

This study has two major strengths and makes two major contributions. First, the study had the participation of a very large number of senior WHO staff with experience with managing various SACs convened by the agency, who together informed our study with a broad range of perspectives. Second, it drew on a well‐established theoretical framework frequently applied in the study of SACs in global environmental governance,^7^ which to our knowledge is yet to be utilized in the study of SACs in global health. The first major contribution is that the study brings insights into a relatively underexplored topic, namely the design and effectiveness of institutions for generating scientific advice in global health. Second, it is a timely contribution to studies about WHO's normative function. WHO recently commissioned its own evaluation of its normative functions.^46^ This evaluation primarily focused on defining and assessing WHO's normative function through a sample of ten normative products, and paid limited attention to the instrumental role SACs play in forming these products. In comparison, our study generates insights about WHO's institutions and processes for generating normative products.

The first main limitation is that this study is primarily informed by, and pools together, the recent general experience of WHO staff who have convened different types of SACs. We have not interviewed stakeholders that have participated in SACs in WHO; these could have offered alternative perspectives of how to achieve quality, relevance, and legitimacy. Moreover, WHO's extensive experience with convening SACs over many decades and the evolution of specific SACs in WHO were not subject to investigation by this study. A consequence of pooling together general insights from across WHO's diverse SACs and not studying the differences between them in greater detail is that this study does not enable judgements about which of WHO's SACs are performing better than others with respect to the principles and design features we identified and the suggestions we have made. Furthermore, the interaction between inputs from SACs and WHO's governing bodies—whose decisions are crucial to the effectiveness of many SACs—have not been explored by this study. These are all areas where future studies could be valuable. Second, while the theoretical frameworks we drew on has its strengths, recent studies have raised questions about the empirical basis for assuming that quality, relevance, and legitimacy represent key determinants of effectiveness. For example, Dunn and Laing recently argued that studies verifying this framework have lacked participation of policymakers from government or industry, and they argued that quality, relevance, and legitimacy poorly reflected what policymakers believed to be the major attributes determining the effectiveness of scientific advice.^43^

## 5 Conclusion

Scientific advisory committees are integral to efforts undertaken by WHO and other UN agencies to bring the latest available scientific evidence to bear on international and national decision‐making in global health. This study identified, articulated, and explored choices when designing SACs that force trade‐offs across quality, relevance, and legitimacy, and identified key design features that all conveners can consider when designing SACs. Overall, paying careful attention to design features affecting transparency, independence, and diversity are likely to be critical for balancing the quality, relevance, and legitimacy of the scientific advice.

## Conflict of Interest

All authors have previously either worked for WHO or served in WHO advisory bodies.

## Supporting information

Supplementary
